# Supplementary material for: Apical Sodium-Dependent Bile Acid Cotransporter, A Novel Transporter of Indocyanine Green, and Its Application in Drug Screening
Source: Int J Mol Sci. 2020 Mar 23;21(6):2202. doi: 10.3390/ijms21062202 (PMC7139337; doi:10.3390/ijms21062202)
Supplement: Supplementary file 1 [file ijms-21-02202-s001.pdf]

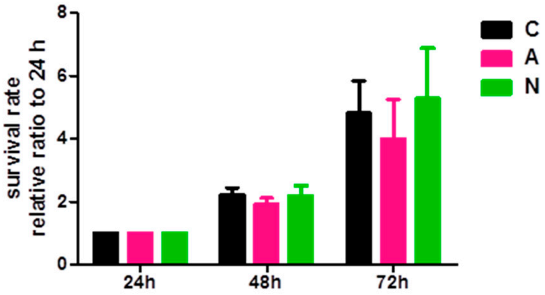

Figure S1 The proliferation observed using the MTT assay in the control and cells expressing ASBT.
